# Soft Sensor Modeling for 3D Transient Temperature Field of Large-Scale Aluminum Alloy Workpieces Based on Multi-Loss Consistency Optimization PINN

**DOI:** 10.3390/s23146371

**Published:** 2023-07-13

**Authors:** Ling Shen, Zhipeng Chen, Xinyi Wang, Jianjun He

**Affiliations:** 1 College of Information Science and Engineering, Hunan Normal University, Changsha 410081, China; shenling@hunnu.edu.cn; 2 School of Automation, Central South University, Changsha 410083, China; xy.wang@csu.edu.cn (X.W.); jjhe@csu.edu.cn (J.H.)

**Keywords:** quenching thermal treatment, temperature soft sensing, physics-informed neural network, loss optimization, partial differential equations

## Abstract

Uniform temperature distribution during quenching thermal treatment is crucial for achieving exceptional mechanical and physical properties of alloy materials. Accurate and rapid prediction of the 3D transient temperature field model of large-scale aluminum alloy workpieces is key to realizing effective thermal treatment. This paper establishes a 3D transient temperature field model of large aluminum alloy workpieces and proposes a multi-loss consistency optimization-based physics-informed neural network (MCO-PINN) to realize soft sensing of the 3D temperature field model. The method is based on a MLP structure and adopts Gaussian activation functions. A surrogate model of the partial differential equation (PDE) is first constructed, and the residuals of the PDE, initial and boundary conditions, and observed data are encoded into the loss functions of the network. By establishing a Gaussian probability distribution model of each loss function and combining it with maximum likelihood estimation, the weight consistency optimization method of each loss function is then proposed to further improve the approximation ability of the model. To optimize the training speed of the network, an adaptive initial-value-eigenvector coding clustering (AIV-ECC) algorithm is finally proposed, which quickly determines the parameters of the Gaussian activation function, reduces the dependence on the initial value and improves the generalization performance of the network. Simulation and industrial experiments demonstrate that the proposed MCO-PINN can solve the 3D transient temperature field model with high precision and high time efficiency based on sparse measurements.

## 1. Introduction

Large-scale aluminum alloy workpieces are the key components of equipment manufacturing in aerospace, new energy industry (such as wind power generation), national defense, and military industry, etc. They are commonly employed in aircraft keel and fuselage load-bearing frames, wind turbine generator frameworks, and end rings of missiles and rockets. These applications demand strict quality control and performance requirements for aluminum alloy materials. Large-scale aluminum alloy workpieces that have undergone effective quenching thermal treatment exhibit significantly enhanced hardness, strength, toughness, and corrosion resistance which can meet the requirements of high-performance, lightweight, and environmentally friendly products of designers and engineers. Therefore, the research on large-scale aluminum alloy workpieces holds immense significance across multiple industries.

The quenching thermal treatment process is a critical step to improve the performance of aluminum alloy workpieces and ensure their safety and reliability [1]. The large-scale vertical quenching furnace is the key equipment in the thermal treatment process. To achieve qualified product quality, the uniformity of temperature distribution in the quenching furnace must be maintained within ±3 °C during the thermal treatment process, which is a great challenge to the control of temperature uniformity in large-scale temperature fields. The real-time temperature field distribution of aluminum alloy workpieces, as the most critical feedback information for the furnace temperature control system, is the core problem of quenching thermal treatment process research.

**Figure 1:**
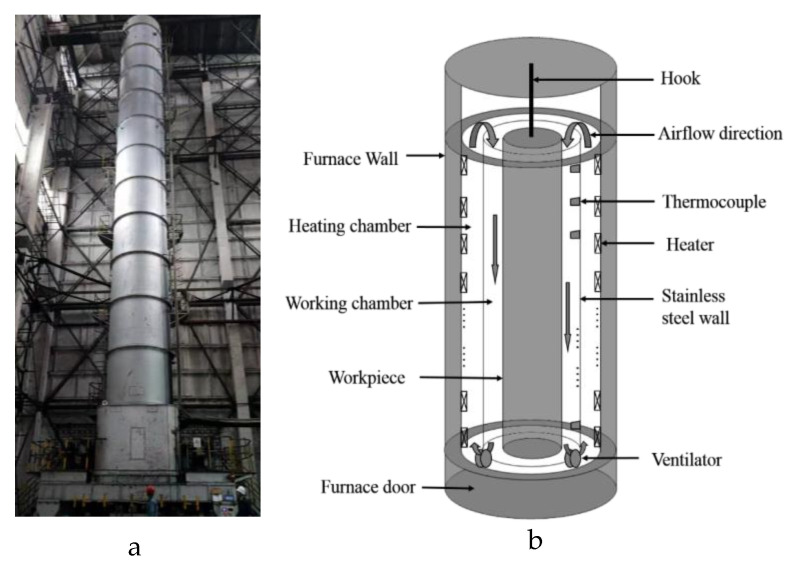
Structure of large-scale vertical quenching furnace (**a**) Physical diagram of quenching furnace. (**b**) Schematic diagram of the furnace structure.

The large-scale vertical quenching furnace is 31 m in height and 3.5 m in radius, as shown in Figure 1. From the outside to the inside, there is the furnace wall, heating chamber, working chamber, and aluminum alloy workpiece. The workpiece is suspended in the center of the workpiece via a hook. The furnace wall consists of slag wool and other insulation materials to reduce heat loss. The heating chamber, equipped with electric heaters, evenly surrounds the lateral wall of the heating chamber from top to bottom. This so-called multi-zone heating method can effectively increase temperature uniformity. The wall of the working chamber, made of stainless steel, aims to further improve the uniformity of the workpiece temperature. Moreover, two high-power ventilators are symmetrically installed at the bottom of the furnace to force air circulation, which enhances convective heat transfer and accelerates the temperature rising velocity. From the structural analysis of the quenching furnace, it can be known that obtaining the real temperature field of the workpiece presents several challenges: (1) The workpiece is suspended in the center of the furnace, and with only a limited number of temperature sensors installed on the inner wall of the working chamber, there exists a significant discrepancy between the measured temperature and the actual workpiece temperature; (2) Multi-zone heating manner and forced air circulation convection are employed in the furnace, which give rise to the coexistence of different heat exchange methods in the thermal treatment process, leading to a strong coupling phenomenon within the furnace; (3) As the temperature increases, hot air in the furnace gradually escapes, causing continuous changes in the furnace’s atmosphere.

Temperature uniformity is a crucial factor in determining the product quality of thermal treatment processes. Numerous research efforts have focused on developing methods for achieving temperature uniformity during thermal treatment processes [2,3]. A temperature prediction model for aluminum alloy workpieces, based on a simplified zone method, has been developed to achieve temperature uniformity distribution in the quenching furnace [4]. In conjunction with the zone method, a heat conduction model for billets affected by radiation and convection was constructed, adopting a suitable convective heat transfer empirical coefficient. This method significantly improves the uniform temperature rise of billet in a large reheating furnace [5]. Peck [6] presented a novel reduced-order transfer-function-based mathematical model for temperature uniformity of a gas powered industrial box furnace, demonstrating that fine adjustments to the controller can reduce temperature rise time while maintaining compliance with Temperature Uniformity Survey (TUS) standards. Xu [7] established a three-dimensional turbulent flow fluid–structure interaction heat transfer model for pit furnaces. By analyzing the flow field and temperature field, the study provides a wall function method to improve the annealing temperature uniformity. Han [8] introduced a comprehensive full-scale industrial reheating furnace model, simulating fuel combustion, flow field, temperature distribution, and slab heating process while emphasizing the significance of skid buttons and dislocated skids. Model predictions align with industrial measurements, demonstrating its applicability for analyzing furnace temperature uniformity distribution. Based on the zone method of radiation analysis, two- and three-dimensional mathematical models for the simulation of the transient thermal behavior in a large bloom reheating furnace were established. The study confirms the feasibility and practicality of zone modeling for integration into a model-based furnace temperature uniformity control system [9]. These studies have significantly contributed to our understanding of temperature uniformity research.

To obtain the workpiece temperature in the thermal treatment process by constructing a mechanism model of the temperature field in the furnace, partial differential equations (PDEs) that describe the thermal treatment process can be solved by numerical methods [10,11,12,13]. The finite element method (FEM) is a numerical technique for solving complex problems in engineering and physics by discretizing large domains into smaller elements. It approximates solutions for partial differential equations (PDEs) by considering the behavior of each element and assembling them into a global system, thus facilitating the analysis of structural, thermal, and fluid dynamics phenomena with increased accuracy and flexibility. Ifis [14] developed an annealing thermal treatment temperature field model for billets and employed FEM for temperature prediction. To analyze the influence of flow field on transient temperature field and smoke emissions, the FEM was employed to explain the dynamic heat transfer effects in various furnace atmospheres influenced by burner angle, preheating temperature, and air–gas ratio [15]. In Refs. [16,17], dynamic boundary conditions within the furnace were described using UDF (User-Defined Function) in the FLUENT platform, enabling the calculation of the entire temperature field and providing guidance for energy-saving production in the thermal treatment process. However, to ensure calculation accuracy, numerical simulation methods can be time-consuming [18,19]. To address this issue, the extrapolation method has been applied to the finite element solution of the transient temperature field model, resulting in significantly improved solution speed and accuracy [20]. The aforementioned methods for solving PDEs of thermal treatment process discretize the solution domain into numerous grids; therefore, coarse grids yield quicker yet less accurate results, while fine grids provide greater accuracy but are slower. Complex PDE systems, such as large-scale thermal treatment processes with stringent temperature uniformity requirements, typically require very fine discretization. As a result, it is very challenging and time-consuming for traditional solvers.

In recent years, the rapid advancement of machine learning has spurred research into utilizing neural network models for solving PDEs [21,22,23]. One approach is data-driven PDE solutions. By using labeled data as input and the exact PDE solution, a neural network can capture nonlinear relationships between labeled data, thereby constructing a data-driven model representing the PDE. A long-proposed, data-driven feed-forward neural network called PDE-NET, which uses Euler discretization for time derivative terms and approximates differential operators via constrained convolution kernels, achieves approximation of partial differential equation systems. Then, the author upgraded PDE-NET to version 2.0, capable of revealing the PDE’s analytical form with minimal prior knowledge while predicting long-term dynamic behavior [24]. Liu [25] discussed the application of a fully connected neural network in function approximation and proposed a universal solver for basic differential equations, which mainly used automatic differentiation to solve the initial and boundary conditions of PDEs. Chen [26] conducted learning and modeling in physical space, using measurement data as nodal values for a deep neural network to approximate unknown PDEs. E. and Han [27] introduced a novel deep learning method for solving high-dimensional PDEs that employs deep learning to approximate gradient operators based on PDE discrete schemes. However, data-driven PDE solution methods are limited by labeled data availability, posing challenges in obtaining sufficient data in some production processes. Transfer learning is a machine learning technique in which a model leverages knowledge gained from one domain to another related domain. By doing so, the model can efficiently learn and adapt to the target task more quickly, benefiting from the experience gained in the source task [28]. In process engineering, transfer learning can be applied to soft sensor design [29]. In Ref. [30], the authors present a domain adaptation extreme learning machine (DAELM) inspired by transfer learning to create a simple soft sensor model for multi-grade chemical processes. An efficient model selection strategy is also developed to refine parameters. By transferring information across operating conditions, DAELM improves prediction accuracy and expands the prediction domain. A new online learning method called JITLTT-MWtr for soft sensor design in modern process industries was proposed in ref. [31]. By combining task transferred just-in-time learning (JITL) and a moving window (MW) learner within a transductive learning setting, the approach leverages transfer learning to effectively address drifts in operating conditions and process characteristics. Easy to implement and robust, JITLTT-MWtr demonstrates high prediction accuracy on multiple datasets, showcasing its potential for industrial applications. Transfer learning reduces training time and dependency on large amounts of labeled data, making it increasingly popular in the industrial application of machine learning techniques in the process engineering domain.

The second approach for solving PDEs involves physics-driven neural networks. This method, which is not constrained by labeled data, exhibits strong generalization capabilities. Sun [32] developed a physics-constrained, data-free deep neural network (DNN) solution for PDEs. By incorporating governing PDEs into the DNN loss and enforcing the initial and boundary conditions through “hard” boundary enforcement, this method effectively enhances the intelligent solution of PDEs under physical constraints. Cai [33] designed an unsupervised deep learning-based numerical approach for approximating PDE solutions via compositional construction, utilizing a first-order system least squares as the loss function to optimize the parameters of the developed neural network. Nonetheless, existing physics-driven methods without labeled data struggle to effectively address unsteady state and source–sink issues.

Utilizing structured prior information to construct a neural network model that incorporates both data and physical information, a physics-informed solution method for PDEs is presented [34]. Physics-informed neural networks (PINNs) have emerged as a powerful paradigm for solving real-life PDE problems [35]. In Ref. [36], a temperature field reconstruction algorithm is proposed based on a PINN-based temperature field inversion method. This method accurately reconstructed the temperature field with limited observations. Combined with a transfer learning strategy and a coefficient matrix condition number based position selection of observations method, the training process was accelerated, and the robustness of the reconstructed model was improved. Zobeiry [37] developed a PINN to solve the heat transfer PDE, and an adaptive normalizing scheme is proposed to reduce errors in loss terms simultaneously. This method has the near real-time simulation capability of problems with any given boundary conditions and is able to predict heat transfer even outside its training zone. In Refs. [38,39], PINN is used to estimate the overall thermal distribution of a transformer. However, the model is one dimensional along the transformer height. In Ref. [40], a PINN is applied to solve the broader aspects of the boundary layer two dimensional flow equations with unbounded domains. For higher-dimensional problems, PINNs are extended to realize three-dimensional fluid temperature and fin temperature prediction without solving the energy equation [41]. Kissas [42] also employed PINNs to recover the entire three-dimensional velocity flow field given four-dimensional flow magnetic resonance imaging data. These studies demonstrate PINN’s potential for solving high-dimensional thermal behavior problems. However, the loss function of PINNs, a fixed weighted combination of the PDE residual term, boundary loss term, and observed data loss term, is susceptible to the weighted combination of competitive multiple loss functions [43]. To improve the accuracy and generalization performance of PINN, it is crucial to optimize loss weight settings.

Based on the analysis of heat transfer mechanism in the quenching furnace, this paper contributes to establish a 3D transient temperature field model of large aluminum alloy workpiece and develop an MCO-PINN to realize soft sensing of the established 3D transient large-scale temperature field model. Firstly, an MLP network is constructed as a surrogate model for solving the PDE of temperature field model, and a Gaussian activation function is chosen to ensure Gaussian distribution in the output of the MCO-PINN. Secondly, the residual of PDE, boundary and initial conditions, and certain measured data are encoded into each loss function of the model. By establishing a Gaussian probability distribution model of each loss function, combined with maximum likelihood estimation, the consistency optimization of the weight of each loss is realized, and the reliability of model prediction is further improved. Thirdly, to improve the convergent speed of the network and expedite training, this study introduces an AIV-ECC algorithm, which rapidly determines the parameters of the MCO-PINN activation function, effectively enhancing the generalization performance of the network, reducing sensitivity to initial values, and increasing the approximation capabilities of the network.

The remainder of the paper is structured as follows. The detailed introduction of the MCO-PINN-based soft sensing method for the 3D temperature field of aluminum alloy workpieces is described in Section 2. The validity and accuracy of the proposed soft sensing method are demonstrated in Section 3 by using the industrial experiment conducted on a 31 m large-scale vertical quenching furnace. The discussion and conclusions are included in Section 4 and Section 5.

## 2. Methods

This section presents the details of the soft sensing framework for the proposed MCO-PINN-based 3D transient temperature field of a large-scale aluminum alloy workpiece, which consists of the establishment of the 3D transient temperature field of the workpiece, the construction of MCO-PINN, and the AIV-ECC algorithm for training optimization.

### 2.1. 3D Transient Temperature Field Modeling for Large-Scale Aluminum Alloy Workpiece

**Figure 2:**
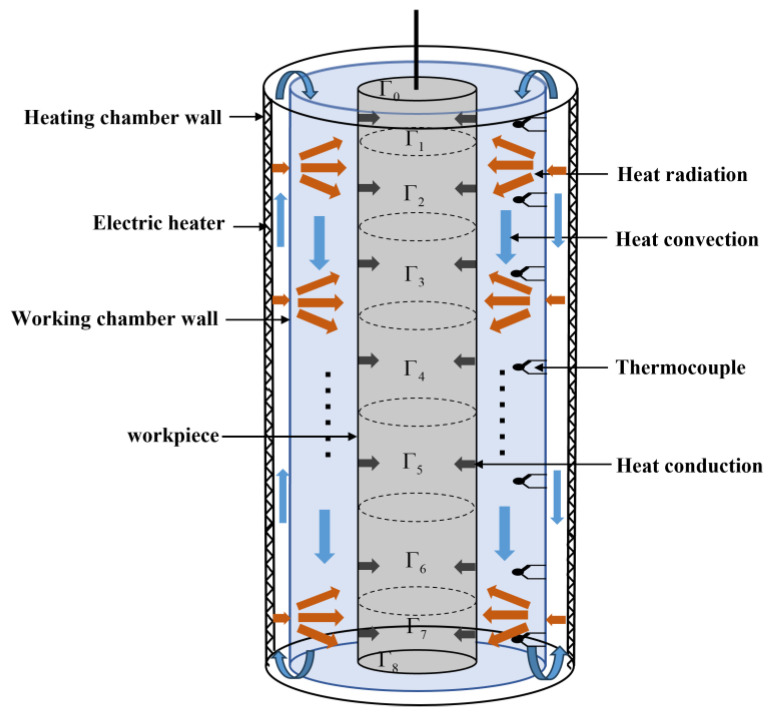
Schematic diagram of heat exchange in the furnace.

During the thermal treatment process, the workpiece is first loaded through the furnace door within 3 to 5 min. Then, the thermal treatment process begins. The thermal treatment process for aluminum alloy workpieces represents an unsteady state heat transfer process, encompassing the internal heat conduction within the workpiece, heat convection between the hot air inside the furnace and the workpiece surface, and heat radiation between the inner wall of the working chamber and the workpiece surface. Figure 2 depicts the schematic diagram of heat exchange in the furnace. In different periods of the thermal treatment process, the heat transfer mechanism is mainly described as

(1) Temperature rising period: During the initial stage of heating, the multi-zone electric heaters operate at full power, rapidly increasing the temperature of the working chamber wall through heat conduction. At this time, there is a significant amount of air inside the furnace, and the ventilators force the air to circulate in the furnace. The heated air transfers heat to the working chamber wall and the workpiece surface, resulting in intense convective heat transfer. Simultaneously, the inner wall of the heating chamber radiate heat onto the working chamber wall, which then radiates heat onto the workpiece surface. After receiving the radiant heat from the inner wall of the working chamber and the convective heat from heated air, the temperature of the workpiece surface rises, and the heat is transferred to the interior through heat conduction, thus realizing the overall temperature rise of the workpiece.
(2) Transition period: At the later period of the temperature rising period, when the furnace temperature approaches the set temperature value, the temperatures of the chamber walls and the workpiece surfaces gradually rise; consequently, the heat radiation effect is rapidly intensified. Simultaneously, the pressure inside the furnace escalates, causing hot air to consistently leak from the furnace door, which results in a substantial decrease in air density and a rapid decline in the heat convection effect.
(3) Temperature holding period: When the temperature in the furnace reaches the temperature set value, the heat treatment enters the temperature holding period. The workpiece temperature is controlled and fluctuates at the temperature set temperature for hours to ensure complete phase change. Generally, the axial temperature distribution uniformity inside the furnace should be within ±3 °C. The idling phenomenon of the ventilators appears within the furnace, making the convective heat exchange nearly negligible. During this period, the surface of the workpiece only receives radiative heat transfer from the working chamber wall, which then transfers inward through heat conduction.

Therefore, it is evident that during the temperature rising and transition periods that the hot air in the furnace transfers heat to the workpiece surface through convective heat transfer, while the inner wall of the working chamber transfers heat to the workpiece surface through radiative heat transfer. The heat is then conducted into the interior of the workpiece. In the temperature holding period, the inner wall of the working chamber transfers heat to the workpiece surface through radiative heat transfer, and then, the heat from the workpiece surface transfers to the inside of the workpiece through heat conduction.

To accurately characterize this process, taking into account the cylindrical geometric structure of both the furnace and the workpiece, a 3D transient temperature field model of a large aluminum alloy workpiece is established as

(1) $$\left\{\begin{array}{l} \frac{\partial^{2}T}{\partial r^{2}}+\frac{1}{r}\frac{\partial T}{\partial r}+\frac{1}{r^{2}}\frac{\partial^{2}T}{\partial \varphi^{2}}+\frac{\partial^{2}T}{\partial z^{2}}=\frac{\rho_{w}c_{w}}{\lambda_{w}}\frac{\partial T}{\partial t}\;\;in\;\;\;\;\;\Omega \\ q_{surface}={\left[\begin{array}{cccc} q_{\Gamma_{0}} & q_{\Gamma_{1}} & \cdots & q_{\Gamma_{8}} \end{array}\right]}^{T}\;\;\;\;\;\;\;\;\;\;\;on\;\;\;\;\;\Gamma \\ q_{\Gamma i}=\;\;\;\left\{\begin{array}{l} q_{ci}\;\;\;\;\;\;\;\;\;\left(0<\bar{T}\leq T_{c}^{sup}\right)\\ q_{ci}+q_{ri}\left(T_{c}^{sup}<\bar{T}\leq T_{r}^{sub}\right)on\;\;\;\;\;\;\Gamma_{i}\\ q_{ri}\;\;\;\;\;\;\;\;\;\left({\bar{T}}_{r}>T_{r}^{sub}\right)\;\;\;\;\;\;i=0,1,\ldots ,8\;\;\;\;\;\; \end{array}\right.\;\;\;\;\;\\ {\left.\frac{\partial T}{\partial z}\right|}_{z=0}=0,{\left.\frac{\partial T}{\partial z}\right|}_{z=l}=0,{\left.T\right|}_{t=0}=T_{0}\;\;\;\;\;\;\;in\;\;\;\;\;\;\;\Omega \;\;\;\;\;\;\;\;\;\;\;\;\;\;\;\;\; \end{array}\right.$$

where T, $\rho_{w}$, $c_{w}$, and $\lambda_{w}$ are the 3D temperature field, density, specific heat capacity, and thermal conductivity of the aluminum alloy workpiece, respectively. r, φ and z represent the radial, circumferential, and axial directions of the workpiece, respectively. $q_{surface}$ is the heat flux imping on the workpiece surface. Γ describes the boundary surface of the workpiece. $q_{c}$ and $q_{r}$ are the convection and radiation heat fluxes impinging on the workpiece surface $q_{surface}$. $T_{0}$ and l are the initial temperature and height of the workpiece. Ω is the computational domain occupied by the workpiece. By solving Equation (1), the temperature values of any position and any time on the exterior or interior of the workpiece during the thermal treatment process can be obtained.

**Figure 3:**
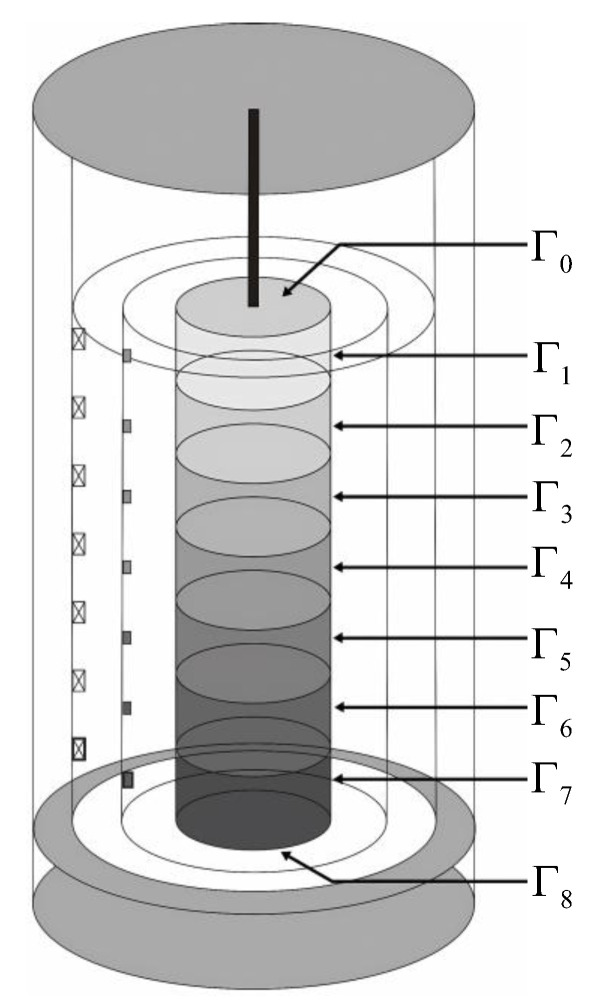
Multi-zone division of the workpiece.

Considering the pronounced regional characteristics of the multi-zone heating method employed in the quenching furnace, wherein the heat fluxes of each heating zone significantly affect the temperature of the workpiece surface closest to them, the outer boundary surface of the workpiece is subdivided into nine sub-surfaces corresponding to the seven thermocouple installation zones on the inner wall of the working chamber. As illustrated in Figure 3, the nine sub-surfaces comprise the top end surface $\Gamma_{0}$, the seven cylindrical toruses $\Gamma_{1}$ to $\Gamma_{7}$, and the bottom end surface $\Gamma_{8}$, respectively. The boundary surface of the workpiece Γ is described as

(2) $$\Gamma =\overset{8}{\underset{i=0}{\cup }}\Gamma_{i}$$

where $q_{\Gamma i}$ is the heat flux imping on the ith sub-surface, which constitutes the convective heat flux $q_{ci}$ and the radiative heat flux $q_{ri}$. $\bar{T}$ represents the average measurement temperatures of seven thermocouples. $T_{c}^{sup}$ is the upper bound temperature when the heat flux imping on the workpiece surface is only provided by convection heat transfer, and $T_{r}^{sub}$ represents the lower bound temperature when the heat flux imping on the forging surface is only provided by radiation heat transfer. The convective heat flux $q_{ci}$ of the ith zone is calculated by the Dittus–Boelter equation, and is expressed by

(3) qci=hi(T−Ti), i=0,1,…,8 hi=0.023⋅kiR⋅Rei0.8⋅Pr0.3Rei=w¯Rρiηi

where $T_{0}=T_{1}\;$ and $T_{7}=T_{8}$. $h_{i}$ is the convection coefficient of the ith sub-surface. For the ith zone, $T_{i}$ is the average temperature of the inner wall of this zone, Pr is the Prandtl number, and R is the radius of the working chamber. $k_{i}$, $\rho_{i}$, $\eta_{i}$, $c_{i}$, Rei, and $\bar{w}$ represent the thermal conductivity, density, dynamic viscosity, specific heat capacity, Reynolds number, and average flow velocity of the air in the ith cylindrical zone. According to the technique setting, $\bar{w}=15\;m/s$. The nonlinear relationship of physical parameters with temperature is obtained through a five-degree polynomial fitting and can be described by Equation (4) [44].

(4) $$\left\{\begin{array}{l} {\left[\begin{array}{ccc} c_{i} & \lambda_{i} & \rho_{i} \end{array}\right]}^{T}=AT+B\\ T={\left[\begin{array}{ccccc} T & T^{2} & T^{3} & T^{4} & T^{5} \end{array}\right]}^{T}\\ A=\left[\begin{array}{ccccc} -3.38 & 6.41\times 10^{-6} & 0 & 0 & 0\\ 1.17\times 10^{-4} & -6.51\times 10^{-8} & 1.98\times 10^{-11} & 0 & 0\\ -0.03 & 7.01\times 10^{-5} & -7.55\times 10^{-8} & 3.85\times 10^{-11} & -7.46\times 10^{-15} \end{array}\right]\\ B={\left[\begin{array}{ccc} 1.0 & -0.0036 & 5.990 \end{array}\right]}^{T} \end{array}\right.$$

The radiation heat flux of the ith sub-surface is $q_{ri}$, which is obtained by

(5) $$q_{ri}=\left\{\begin{array}{l} Q_{i}/S_{i}\;,i=1,\cdots ,7\\ 0\;\;\;\;,\;\;\;\;\;i=0,8 \end{array}\right.$$

$S_{i}$ and $Q_{i}$ are the area and net radiation heat flux of the ith cylindrical torques. $Q_{i}$ can be calculated by

(6) $$\left\{\begin{array}{l} Q=(\bar{SS}-diag\left\{S\right\}diag\left\{\varepsilon \right\})E\\ \bar{SS}=diag\left\{S\right\}diag\left\{\varepsilon \right\}{\left[diag\left\{S\right\}\;-\;\;\;\bar{ss}(I-diag\left\{\varepsilon \right\})\right]}^{-1}\bar{ss}diag\left\{\varepsilon \right\}\\ \bar{s_{i}s_{j}}=\frac{R^{4}}{\pi }\int_{\theta_{i}}\int_{l_{i}}\int_{\theta_{j}}\int_{l_{j}}\frac{\left[1-\cos(\theta_{i}-\theta_{j})\right]}{d^{4}}d\theta_{i}dl_{i}d\theta_{j}dl_{j} \end{array}\right.$$

where $S={\left[s_{i}\right]}^{T}$ is the area vector of the torus, $\varepsilon ={\left[\varepsilon_{i}\right]}^{T}$ is the surface emissivity, $E={\left[E_{i}\right]}^{T}={\left[\sigma T_{i}\right]}^{T}$ describes the blackbody emissive power, and $Q={\left[Q_{i}\right]}^{T}$ is the net radiation flux on the lateral surface of the workpiece. $\bar{ss}={\bar{ss}}^{T}=[\bar{s_{i}s_{j}}]$ and $\bar{SS}={\bar{SS}}^{T}=[\bar{S_{i}S_{j}}]$ are the direct exchange area and total exchange area between surface to surface, where the center of the two infinitesimal sections on the surface are i and j. $\theta_{i}$ and $\theta_{j}$ are the angles of the light ray and the perpendicular to the surface section. $l_{i}$ and $l_{j}$ are the heights of the infinitesimal surfaces. According to Equation (6), total exchange area $\bar{SS}$ is the key to obtain the net radiative heat flux Q. $\bar{SS}$ is only concerned with the geometry of the furnace, which is independent of temperature, so it is only required to be calculated once.

### 2.2. Solution of 3D Transient Temperature Field Model of Workpiece Based on MCO-PINN

**Figure 4:**
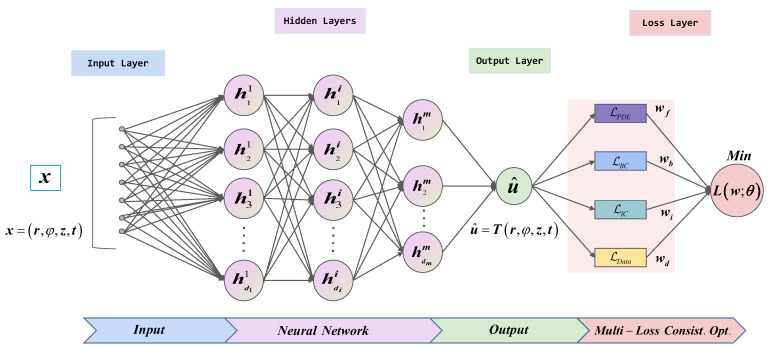
Framework of MCO-PINN.

The proposed framework for MCO-PINN is depicted in Figure 4. Firstly, a multi-layer MLP model is employed to construct a deep neural network with an output $\hat{u}$, which serves as a surrogate model for the solution of the 3D transient temperature field model for the workpiece. The input x of MCO-PINN is the spatial and temporal information (r, φ, z, t), while the output $\hat{u}$ is the temperature information T (r, φ, z, t). The residual of the PDE, boundary and initial conditions, and certain measured data are set as constraints and encoded into the loss functions $L_{PDE}$, $L_{BC}$, $L_{IC}$, and $L_{Data}$ of the deep neural network for training. During training, a Gaussian probability distribution model is established for each loss function, combined with the maximum likelihood estimation method, and the weight of each loss ($w_{f},w_{b},w_{i}$ and $w_{d}$) is adaptively adjusted. This approach transforms the global minimization of the loss objective function for the network into a process of synchronously and consistently minimizing multiple losses, thereby balancing the backpropagation gradient size of the residual term, boundary loss term, and data loss term in the loss function. As a result, gradient disappearance and explosion are averted, and the MCO-PINN solution accuracy is improved. Finally, using the powerful nonlinear black box approximation, massive data fitting, and fast calculation abilities of deep neural networks, the problems faced by traditional numerical methods in solving high-dimensional PDE model, such as low solution accuracy, difficult inversion of model parameters, high data sensitivity, poor adaptation ability of new data, and time-consuming solution in large-scale domains are solved. Specifically:

The solution of the PDE model (1) is $\hat{u}(x)$ in $\Omega \subset {\mathbb{R}}^{d}$, where $x=\left(x_{1},\cdots ,x_{d}\right)$. Then, the PDE and boundary conditions shown in Equation (1) can be rewritten into the general form as

(7) $$f\left(x;\;\frac{\partial u}{\partial x_{1}},\cdots ,\frac{\partial u}{\partial x_{d}},\;\frac{\partial^{2}u}{\partial x_{1}\partial x_{1}},\cdots ,\frac{\partial^{2}u}{\partial x_{d}\partial x_{d}};\lambda \right)=0,\;\;x\in \Omega$$

(8) $$B\left(u,x\right)=0\;,\;\;x\in \partial \Omega$$

The MLP model of m full connection layers is constructed as

(9) $$N_{\theta }=L_{m}\circ \sigma \circ L_{m-1}\circ \sigma \circ \cdots \circ \sigma \circ L_{1}$$

and

(10) $$\left\{\begin{array}{l} L_{1}\left(x\right)=W_{1}x+b_{1},\;\;\;\;\;\;\;\;\;W_{1}\in {\mathbb{R}}^{d_{1}\times d},\;\;\;\;\;\;\;\;\;b_{1}\in {\mathbb{R}}^{d_{1}}\\ L_{i}\left(x\right)=W_{i}x+b_{i},\;\;\;\;\;\;\;\;\;\;W_{i}\in {\mathbb{R}}^{d_{i}\times d_{i-1}},\;\;\;\;\;\;\;b_{i}\in {\mathbb{R}}^{d_{i}},\;\;\;\forall i=2,3,4,\cdots ,m \end{array}\right.$$

where $W_{i}$ and $b_{i}$ are the weights and biases at the ith layer. Let the set of all weight matrices and bias vectors be the following

(11) $$\theta ={\left\{W_{i},b_{i}\right\}}_{i=1,2,\cdots ,m}$$

Apparently, the size and depth of the deep neural network are ${\left\|\theta \right\|}_{l^{0}}$ and m.

To evaluate the deviation between the output $\hat{u}$ of the depth neural network and the constraints of the PDE model, a loss function is defined as

(12) $$\begin{array}{l} min_{\theta ,\lambda }L\left(\theta ,\lambda ;T\right)=w_{f}L_{PDE}\left(\theta ,\lambda ;T_{f}\right)+w_{i}L_{IC}\left(\theta ,\lambda ;T_{i}\right)\\ \;\;\;\;\;\;\;\;\;\;\;\;\;\;\;\;\;\;\;\;\;\;\;\;\;\;\;\;+w_{b}L_{BC}\left(\theta ,\lambda ;T_{b}\right)+w_{d}L_{Data}\left(\theta ,\lambda ;T_{data}\right) \end{array}$$

with

(13) $$\left\{\begin{array}{l} L_{PDE}\left(\theta ,\lambda ;T_{f}\right)=\frac{1}{\left|T_{f}\right|}\sum_{x\in T_{f}}{\left\|f\left(x;\;\;\;\frac{\partial u}{\partial x_{1}},\cdots ,\frac{\partial u}{\partial x_{d}},\;\frac{\partial^{2}u}{\partial x_{1}\partial x_{1}},\cdots ,\frac{\partial^{2}u}{\partial x_{d}\partial x_{d}};\lambda \;\;\right)\right\|}_{2}^{2}\\ L_{IC}\left(\theta ,\lambda ;T_{i}\right)=\frac{1}{\left|T_{i}\right|}\sum_{x\in T_{i}}{\left\|\hat{u}\left(x\right)-u\left(x\right)\right\|}_{2}^{2}\\ L_{BC}\left(\theta ,\lambda ;T_{b}\right)=\frac{1}{\left|T_{c}\right|}\sum_{x\in T_{b}}{\left\|B\left(\hat{u},x\right)\right\|}_{2}^{2}\\ L_{Data}\left(\theta ,\lambda ;T_{data}\right)=\frac{1}{\left|T_{data}\right|}\sum_{x\in T_{data}}{\left\|\hat{u}\left(x\right)-u\left(x\right)\right\|}_{2}^{2} \end{array}\right.$$

where $w_{f},w_{b},w_{i}$, and $w_{d}$ describe the weight of each loss. $T_{i},T_{b},T_{data}$ are the number of residual points of the initial, boundary and experimental measured values of the PDE model, respectively. $T_{f}$ represents a set of predefined points to measure the matching degree between the output $\hat{u}$ of the deep network and the PDE model. $\lambda =\left(T_{c}^{sup},T_{r}^{sub}\right)$ is the parameter vector to be identified in the model.

To achieve multi-loss consistency optimization of the MCO-PINN, assume that the multiple outputs of the network conform to a Gaussian distribution, with the mean value of maximum likelihood estimation being $\hat{u}$. $\hat{u}$ is an approximation of the true value u of the solution to the PDE model. The variance of maximum likelihood estimation is $\varsigma_{d}$ which is an uncertainty parameter and described as

(14) $$p\left(u|\hat{u}\left(x;\theta \right)\right)=N\left(\hat{u}\left(x;\theta \right),\varsigma_{d}\right)$$

The maximum likelihood function is used to optimize the uncertain parameter, yielding

(15) $$-\log p\left(u\left|\hat{u}\left(x;\theta \right)\right.\right)\propto \frac{1}{2\varsigma_{d}^{2}}{\left\|u-\hat{u}\left(x;\theta \right)\right\|}^{2}+\log\varsigma_{d}=\frac{1}{2\varsigma_{d}^{2}}L_{Data}\left(\theta ,\lambda \right)+\log\varsigma_{d}$$

Taking into account that the mean of the maximum likelihood estimation $\hat{u}$ is affected by the boundary and initial values and the residual of the PDE model, Equation (15) can be extended to a multi-output neural network and is expressed as

(16) $$\begin{array}{l} -\log p\left(f,i,b,d\left|\hat{u}\left(x;\theta \right)\right.\right)\propto \frac{1}{2\varsigma_{f}^{2}}{\left\|f-\hat{u}\left(x;\theta \right)\right\|}^{2}+\frac{1}{2\varsigma_{i}^{2}}{\left\|i-\hat{u}\left(x;\theta \right)\right\|}^{2}\\ \;\;\;\;\;\;\;\;\;\;\;\;\;\;\;\;\;\;\;\;\;\;\;\;\;\;\;\;\;\;\;\;\;\;\;\;\;\;\;\;\;\;+\frac{1}{2\varsigma_{b}^{2}}{\left\|b-\hat{u}\left(x;\theta \right)\right\|}^{2}\;\;+\frac{1}{2\varsigma_{d}^{2}}{\left\|d-\hat{u}\left(x;\theta \right)\right\|}^{2}\\ \;\;\;\;\;\;\;\;\;\;\;\;\;\;\;\;\;\;\;\;\;\;\;\;\;\;\;\;\;\;\;\;\;\;\;\;\;\;\;\;\;\;+\log\varsigma_{f}\varsigma_{i}\varsigma_{b}\varsigma_{d} \end{array}$$

Therefore, the optimization objective of loss function of MCO-PINN can be written as follows:

(17) $$\begin{array}{l} min_{\theta ,\lambda }L\left(\theta ,\lambda ;T\right)=\frac{1}{2\varsigma_{f}^{2}}L_{PDE}\left(\theta ,\lambda ;T_{f}\right)+\frac{1}{2\varsigma_{i}^{2}}L_{IC}\left(\theta ,\lambda ;T_{i}\right)\\ \;\;\;\;\;\;\;\;\;\;\;\;\;\;\;\;\;\;\;\;\;\;\;\;\;\;\;\;\;+\frac{1}{2\varsigma_{b}^{2}}L_{BC}\left(\theta ,\lambda ;T_{b}\right)+\frac{1}{2\varsigma_{d}^{2}}L_{Data}\left(\theta ,\lambda ;T_{data}\right)+\log\varsigma_{f}\varsigma_{i}\varsigma_{b}\varsigma_{d} \end{array}$$

where $\varsigma =\left\{\varsigma_{f},\varsigma_{i},\varsigma_{b},\varsigma_{d}\right\}$ represents the adaptive weight of each loss term. $w=\left\{w_{f},w_{b},w_{i},w_{d}\right\}$ with $w=\frac{1}{2\varsigma^{2}}$ demonstrate the total weights of loss terms. The goal of MCO-PINN training is to find the best model parameters $\theta^{*}$ and appropriate adaptive weights $\varsigma^{*}$ to minimize the loss $L\left(\theta ,\lambda ;T\right)$.

### 2.3. Training Optmization of MCO-PINN

To achieve multi-loss consistency optimization in MCO-PINNs, an assumption must be satisfied; that is, the various network outputs should conform to Gaussian distribution requirements. To comply with this stringent assumption, the radial basis activation function has been chosen as the activation function of the network. This selection ensures that the output of MCO-PINN exhibits a Gaussian distribution, which is achieved through the linear superposition of Gaussian functions. $\sigma_{j}^{i}$ represents the activation function of the jth hidden node in the ith hidden layer which is described as

(18) $$\sigma_{j}^{i}\left(x\right)=exp\left(-\frac{{\left\|x-\mu_{j}^{i}\right\|}^{2}}{\delta_{j}^{i}{}^{2}}\right),\;\;\;i=1,2,3,\cdots ,m,\;\;\;\;j=1,2,3,\cdots ,d_{m}$$

The center $\mu_{j}^{i}$ and width $\delta_{j}^{i}$ of the activation function serve as structural parameters of MCO-PINN, which can be predetermined through pre-training. Generally, high-precision clustering algorithms can efficiently uncover the intrinsic relationships within training sample data, enabling the determination of MCO-PINN activation function parameters and effectively enhancing the network’s generalization performance. However, traditional distance-based clustering algorithms necessitate multiple iterations, leading to reduced efficiency and heightened sensitivity to initial values. Consequently, the AIV-ECC algorithm, outlined as follows, is proposed.

An AIV-ECC algorithm is introduced to explore the inherent clustering features and correlation of input samples, which is designed to enhance the network’s capacity for accurately representing the fundamental characteristics of the underlying data. By determining a clustering number k, the input sample matrix X is represented as

(19) $$XE=\left[X_{1},\cdots ,X_{i},\cdots ,X_{k}\right],\;\;\;\;\;\;X_{i}=[x_{{}_{1}}^{\left(i\right)},\cdots ,x_{m_{i}}^{\left(i\right)}]$$

where E is the rotation matrix. $X_{i}$ is a $n\times m_{i}$ matrix which represents the samples in the ith cluster, and $m_{i}$ is the number of samples in the ith cluster. The correlation square sum cost function of the partition ∏ of the matrix X is expressed as

(20) $$ss\left(\prod \right)=\sum_{i=1}^{k}\sum_{m=1}^{m_{i}}{\left\|x_{m}^{\left(i\right)}-{\bar{x}}_{i}\right\|}^{2},{\bar{x}}_{i}=\sum_{m=1}^{m_{i}}\frac{x_{m}^{\left(i\right)}}{m_{i}}$$

where ${\bar{x}}_{i}$ is the average of the samples in the ith cluster. Introducing a vector $e=(\overset{m_{i}}{\overset{︷}{1,\cdots 1,\cdots ,1}})$, and the Hilbert–Schmidt norm ${\left\|X\right\|}_{F}=\sqrt{Tr\left(X^{T}X\right)}$, Equation (20) is rewritten as

(21) $$ss\left(\prod \right)=\sum_{i=1}^{k}{\left\|X_{i}-{\bar{x}}_{i}e^{T}\right\|}_{F}^{2}=\sum_{i=1}^{k}{\left\|X_{i}\left(I_{mi}-ee^{T}/m_{i}\right)\right\|}_{F}^{2}$$

${\left(I_{mi}-ee^{T}/m_{i}\right)}^{2}=I_{mi}-ee^{T}/m_{i}$ is the projection matrix. We set an orthogonal matrix A of m×k as

(22) $$A=\begin{array}{c} m_{1}\\ m_{2}\\ \vdots \\ m_{k} \end{array}\left[\begin{array}{cccc} \frac{e}{\sqrt{m_{1}}} & & & \\ & \frac{e}{\sqrt{m_{2}}} & & \\ & & \ddots & \\ & & & \frac{e}{\sqrt{m_{k}}} \end{array}\right]$$

Equation (21) is simplified as

(23) $$ss\left(\prod \right)=Tr\left(X^{T}X\right)-Tr\left(A^{T}X^{T}XA\right)$$

It is evident that the optimal approach for partitioning X into k clusters can be achieved by employing the partition strategy, which entails minimizing the correlation square sum cost function $min\;\;\;\;ss\left(\prod \right)$ in Equation (23). Given that $Tr\left(X^{T}X\right)$ is determined by the sample space, the problem of $min\;\;\;\;ss\left(\prod \right)$ is considered as an equivalent optimization problem described as

(24) $$\underset{U^{T}U=I_{k}}{max}\;\;\;\;\;\;\;Tr\left(A^{T}X^{T}XA\right)$$

According to the Ky Fan theorem, when dealing with a real and symmetric matrix $B=X^{T}X$ with eigenvalues $\lambda_{1}\geq \lambda_{2}\geq \cdots \lambda_{m}$ and eigenvectors $\lambda_{1}\geq \lambda_{2}\geq \cdots \lambda_{m}$, Equation (25) holds constant under the constraint of $A^{T}A=I_{k}$.

(25) $$\lambda_{1}+\lambda_{2}+\cdots \lambda_{k}=\underset{U^{T}U=I_{k}}{max\;}\;\;\;Tr\left(A^{T}BA\right)\;$$

The optimal matrix is $U^{*}=\left[\nu_{1},\cdots ,\nu_{k}\right]H$, and H is any orthogonal matrix.

To quickly obtain $U^{*}$ and k, we can assume that each submatrix $X_{i}$ corresponds to an optimal class. Given this assumption, Equation (26) holds.

(26) $$X^{T}X=\left[\begin{array}{cccc} X_{1}^{T}X_{1} & 0 & \cdots & 0\\ 0 & X_{2}^{T}X_{2} & \cdots & 0\\ \vdots & \vdots & \ddots & \vdots \\ 0 & 0 & \cdots & X_{k}^{T}X_{k} \end{array}\right]$$

The largest eigenvector of $X_{i}^{T}X_{i}$ is set as $y_{i}$, and $Y_{k}$ is constructed as

(27) $$Y_{k}=\begin{array}{c} m_{1}\\ m_{2}\\ \vdots \\ m_{k} \end{array}\left[\begin{array}{cccc} y_{1} & & & \\ & y_{2} & & \\ & & \ddots & \\ & & & y_{k} \end{array}\right]$$

According to the $Davis-Kahan\;sin\left(\Theta \right)$ theorem, by ignoring the higher order term, Equation (28) can be deduced and expressed as

(28) $$R_{k}^{T}=V^{T}Y_{k}^{T}$$

$V=\left[v_{1},\cdots ,v_{k}\right]$ is an k×k orthogonal matrix. Expanding Equation (28) as the following

(29) $$R_{k}^{T}=(\underset{cluster\;\;\;1}{\underset{︸}{y_{11}v_{1},\cdots ,y_{1m_{1}}v_{1}}},\cdots ,\underset{cluster\;\;\;k}{\underset{︸}{y_{k1}v_{k},\cdots ,y_{km_{k}}v_{k}}})$$

Equation (28) indicates that the optimal categorization approach for input sample matrix X can be determined through the orthogonal linear transformation of matrix $R_{k}^{T}$, which consists of the first k maximum eigenvectors of matrix $X^{T}X$. The QR decomposition of matrix $R_{k}^{T}$ can be used to facilitate the orthogonal transformation of matrix $R_{k}^{T}$ to $Y_{k}^{T}$.

(30) $$R_{k}^{T}P=QR=Q\left[R_{11},R_{12}\right]$$

where P represents a permutation matrix, Q is an k×k orthogonal matrix, and $R_{11}$ is a k×k upper triangular matrix. Based on the calculation of $R_{k}^{T}$, the cluster discrimination matrix $\overset{⌢}{R}$ is calculated by

(31) $$\overset{⌢}{R}=R_{11}^{-1}\left[R_{11},R_{12}\right]P^{T}=\left[I_{k},R_{11}^{-1}R_{12}\right]P^{T}$$

The cluster category for each data vector can be determined by identifying the index of the element with the largest absolute value in the corresponding column of matrix $\overset{⌢}{R}$. Subsequently, we can obtain the sample set $I\left(C_{i}\right)$ for any category i and compute the cluster center vector $c_{i}$ using the following equation:

(32) $$c_{i}={\sum_{x_{m}\in I(C_{i})}x_{m}/m_{i}}_{i=1,2,\cdots ,k}$$

where $m_{i}$ represents the number of elements in set $I\left(C_{i}\right)$. The width of the cluster $\vartheta_{i}$ is defined as the dispersion degree of each cluster sample relative to the cluster center vector and is calculated by

(33) $$\vartheta_{i}=\frac{2max{\left\{{\left\|x_{i}\left(k\right)-c_{i}\right\|}^{2}\right\}}_{\left\{x_{i}\left(k\right)\in I(C_{i})\right\}}}{3}$$

The detailed steps of AIV-ECC algorithm are as follows:

***Step* 1:** For the existing samples $X=\left\{x_{1},x_{2},\ldots ,x_{m}\right\}$, randomly re-sample N times to form the bootstrap samples $X^{*}=\left\{x_{1}^{*},x_{2}^{*},\ldots ,x_{m}^{*}\right\}$ and calculate the sum of within-class distances of each bootstrap sample according to the following equation. Then, the statistics of N bootstrap samples of these N times are obtained and arranged from small to large.

(34) $$D^{*}(k)=\sum_{q=1}^{k}\frac{1}{n_{q}}\sum_{i,j\in C_{q}}\left(\max\left\{\left|x_{i}-x_{j}\right|\right\}\right)$$

where $Cluster=\left\{C_{q},q=1,2,3,\ldots ,k\right\}$. Initialize k=2;

***Step* 2:** Since the N bootstrap statistics are normally distributed, the confidence interval under the confidence degree (1−α) can be obtained $\left[D_{\frac{\alpha }{2}}^{*}(k),D_{1-\frac{\alpha }{2}}^{*}(k)\right]$;

***Step* 3:** The data sample *X* is clustered by ECC clustering algorithm to obtain the k clusters of data sample, the clustering center vector matrix $c=\left\{c_{1},c_{2},\cdots c_{k}\right\}$, and the clustering width vector $\vartheta =\left\{\vartheta_{1},\vartheta_{2},\cdots ,\vartheta_{k}\right\}$ at this time;

***Step* 4:** Given the confidence level α, if ω(k) of the clustered samples under k classes is within the confidence interval of $\left[D_{\frac{\alpha }{2}}^{*}(k),D_{1-\frac{\alpha }{2}}^{*}(k)\right]$, then end the AIV-ECC algorithm; otherwise, k=k+1.

***Step* 5:** Repeat Step 3 and Step 4.

The pseudo code for MCO-PINN training solution is as follows in Algorithm 1:
**Algorithm 1** Train MCO-PINN**1: Data:** Historical training sample set $X={\left\{x_{1},\cdots x_{i},\cdots ,x_{n}\right\}}_{x_{i}\in {\mathbb{R}}^{d}}$; Training sample sets satisfy the true value, initial value, boundary value, and parameter identification value of the 3D transient workpiece temperature field model $T_{f},T_{i},T_{b},T_{data}$;
**2: Initialize network depth:** Cluster the sample set X with the AIV-ECC algorithm and determine the depth of the network as the clustering number m; Assign the training samples as $X=\left[X_{1},\cdots ,X_{i},\cdots ,X_{m}\right]$;
**3: Initialize network size:** Cluster $X_{i}$ with the AIV-ECC algorithm and determine the number of hidden nodes in the ith layer as the number of clusters $k^{i}$, the clustering center vector matrix $c^{i}=\left\{c_{{}_{1}}^{i},c_{{}_{2}}^{i},\cdots c_{k}^{i}\right\}$ and the cluster width vector $\vartheta^{i}=\left\{\vartheta_{{}_{1}}^{i},\vartheta_{{}_{2}}^{i},\cdots ,\vartheta_{{}_{k}}^{i}\right\}$; Initialize the radial basis activation functions for all hidden nodes;
**4: Initialize training parameters:** Randomly initialize network parameters and weights λ, ς and θ;
**5: Input:** Input current sample vector $x=\left(x_{1},\cdots ,x_{d}\right)$;
**6: Construct Gaussian probability model:** Construct Gaussian probabilistic models with mean given by the output of PINNs and the adaptive weight collection ς;
**7: Train:** Use K gradient descent iterations to update the parameters ς and θ as
**For**
k=1     to    K    do         (a)$\mathrm{Define}\;\mathrm{the}\;\mathrm{adaptive}\;\mathrm{weighted}\;\mathrm{loss}\;\mathrm{function}\;L\left(\theta ,\lambda ;T\right)$ (Equation (17)) based on the maximum likelihood estimation.         (b)Tune the adaptive weight via Adam optimizer to maximize the probability of meeting constraints.$\;\;\;\;\;\;\;\;\;\;\;\;\;\;\;\;\;\;\;\;\;\;\;\;\;\;\;\;\;\;\;\;\;\;\;\;\;\;\;\varsigma_{k+1}\leftarrow Adam\left(L\left(\theta ,\lambda ;T\right)\right)$         (c)Update the network weight θ via Adam optimizer.$\;\;\;\;\;\;\;\;\;\;\;\;\;\;\;\;\;\;\;\;\;\;\;\;\;\;\;\;\;\;\;\;\;\;\;\;\;\;\;\theta_{k+1}\leftarrow Adam\left(L\left(\theta ,\lambda ;T\right)\right)$
**End for** **8: Output**         The best model with parameters $\theta^{*}$ and the final adaptive weight $\varsigma^{*}$.

## 3. Results

**Figure 5:**
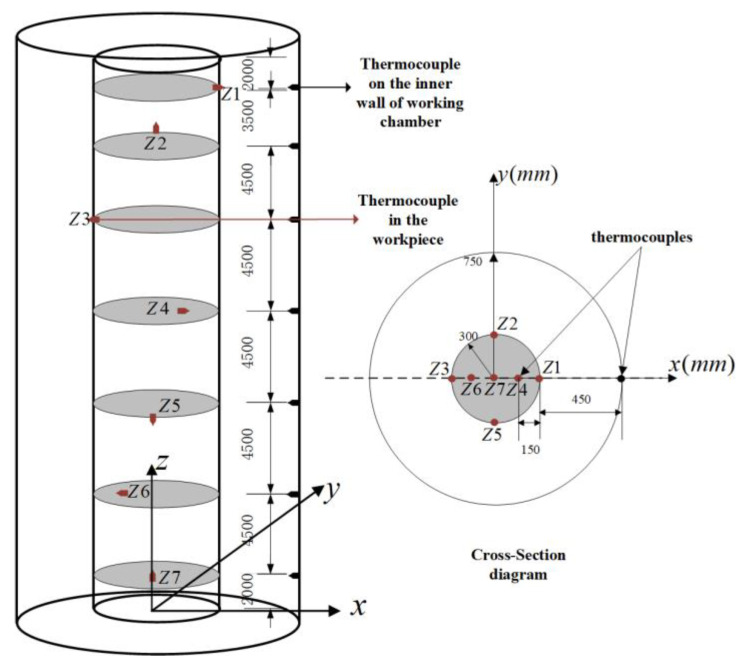
Sketch of the location of the workpiece thermocouples.

Industrial experiments were conducted in a 31 m large-scale vertical quenching furnace. The specification of the experimental aluminum alloy workpiece is 360X35, with the alloy state 6061T6511 and batch number CJ2025; the gradient temperature setpoints are 465 °C, 500 °C, and 530 °C. The physical structure of the workpiece is 0.3 m in radius and 30 m in length. To obtain the surface and interior workpiece temperature, thermocouples were installed at different positions on the surface and inside of the experimental workpiece. The sketch of the installation location of the thermocouples is shown in Figure 5. To collect more temperature information from the workpiece using a limited number of thermocouples, seven thermocouples from Zone 1 to Zone 7 were installed in the industrial experiment. In the sketch, these zones are abbreviated as Z1–Z7. Thermocouples Z1, Z2, Z3, and Z5 are situated on the workpiece surface, while Z4 and Z6 are positioned at half of the workpiece radius. Z7 is placed at the center of the workpiece. The cross-sectional diagram depicts the distance of each thermocouple from its corresponding location to the center of the workpiece.

In this experiment, 3030 datasets were collected at a sampling interval of 10 s. Temperature data for parameter identification of the 3D temperature field model were obtained by installing thermocouples on the experimental workpiece, and there are 510 sets of measured temperature data. Therefore, the training set for $T_{f}$ is the 3030 sets of internal thermocouple measurements of the workpiece, and each is represented as $\left(T_{Z4},T_{Z6},T_{Z7}\right)$. $T_{i}$ is the initial workpiece temperature and it is a fixed temperature set. The training set for $T_{b}$ is the 3030 sets of thermocouple measurements of the workpiece surface, and each is represented as $\left(T_{Z1},T_{Z2},T_{Z3},T_{Z5}\right)$. $T_{data}$ is used to identify the parameters $\lambda =\left(T_{c}^{sup},T_{r}^{sub}\right)$. Therefore, the datasets specially used for model parameter identification in the experiment are 510 sets of thermocouple measurements, and each data vector is represented as $\left(T_{Z1},T_{Z2},T_{Z3},T_{Z4},T_{Z5},T_{Z6},T_{Z7}\right)$.

The simulation test platform executes the system on a machine equipped with an NVIDIA RTX 4090 GPU and Windows OS. The developed MCO-PINN was implemented using Python. The MCO-PINN contains 6 hidden layers with 128, 128, 64,64, 32, and 32 nodes in each layer. There are 3540 training samples were used, encompassing 295 batches with a batch size of 12, resulting in 8850 iterations and 30 epochs. To ensure that the ANN model has good generalization ability, we utilized five-fold cross-validation.

**Figure 6:**
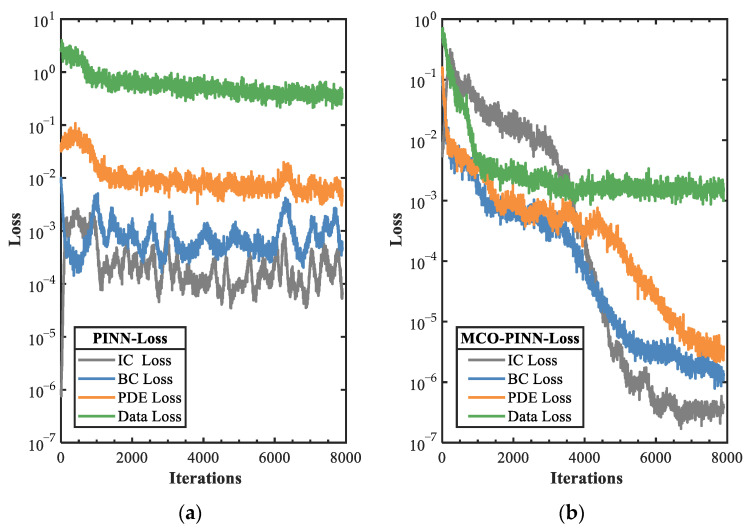
Evolution of each loss function (**a**) PINN. (**b**) MCO-PINN.

Figure 6 compares the iteration convergence curves of the error functions for both PINN and MCO-PINN. During the model training process, for the PINN network, we artificially assigned a fixed weight vector $w=\left\{w_{f}=0.25,w_{b}=0.25,w_{i}=0.25,w_{d}=0.25\right\}$, remaining constant throughout the training process. However, within the MCO-PINN, due to its adaptive weight adjustment, we designated an initial value for the weight vector as $w=\left\{w_{f}=0.5,w_{b}=0.25,w_{i}=0.5,w_{d}=0.25\right\}$. As the weights adaptively adjusted, the network training loss stabilized; notably, this occurred when the network training iterations exceeded 8000 steps. The fluctuation ranges of the various weight values were as follows: $w_{f}=0.7586\pm 0.5888$, $w_{b}=0.09349\pm 0.07839$, $w_{i}=0.09318\pm 0.29138$, and $w_{d}=0.05473\pm 0.04143$. As observed in Figure 6a, during the first 1000 iterations for the PINN, the PDE residual loss and initial condition loss increase while boundary loss and data loss decline. As the number of iterations increases, reaching 8000 steps, the PDE loss, IC loss, BC loss, and data loss drop to (10^−2^, 10^−4^, 10^−3^, 10^0^), respectively. Figure 6b illustrates that employing the MCO-PINN method results in consistent declines for all loss functions due to the proposed consistency optimization method. It is particularly notable after 3000 iterations when rapid, consistent convergence is achieved, with PDE loss, IC loss, and BC loss all converging to levels below 10^−5^. Data loss rapidly converges initially. As the iteration step increases, it does not display rapid declines, ensuring the structural stability of the PDE. Based on the iteration convergence curves presented in both Figure 6a,b, the MCO-PINN not only exhibits faster convergence speed than PINNs, accelerating the solution speed of the network, but also demonstrates superior accuracy relative to PINNs.

**Figure 7:**
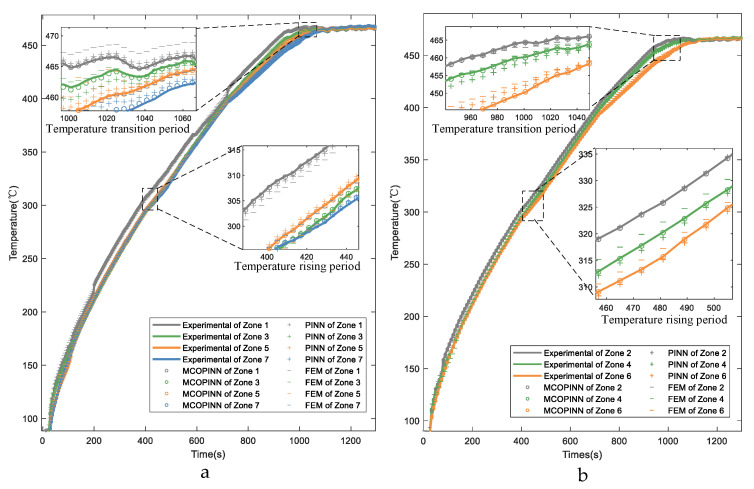
Comparisons of the workpiece temperature with different methods in the temperature rising and transition periods (**a**) Zones 1, 3, 5, and 7. (**b**) Zones 2, 4, and 6.

To verify the effectiveness of MCO-PINN in soft sensing of 3D temperature field, MCO-PINN, PINN, and FEM are employed to solve the 3D temperature field model of the workpiece, and the workpiece temperature curves are shown in Figure 7. As the highly uniform distribution of the large-scale temperature field inside the furnace is a critical factor for ensuring the effectiveness of quenching thermal treatment, the temperature distribution of the seven measurement points in the workpiece exhibits strong consistency, with only minor deviations. To provide a more intuitive and clear comparison of the results, the paper divides the seven zones of the workpiece into three representative positions (upper, middle, and lower) for regional comparison. The temperature curves during the rapid heating and transition periods are illustrated in Figure 7.

Observing the enlarged images during the temperature rising period in Figure 7a,b, it is evident that the furnace temperature increases rapidly by 15 °C within the 50 s, and noticeable temperature gradients emerge across various zones of the workpiece. During this period, abundant hot air circulates within the furnace, causing intense convective heat exchange, which leads to rapid heating. As the air heats up, it rises and accumulates in the upper and middle parts of the furnace. Although the ventilators at the furnace bottom force air circulation, because the quenching furnace is as high as 31 m, Zone 1 is close to the top of the furnace, and Zone 7 is located at the lower part of the furnace and close to the bottom door; the temperature from Zone 1 to Zone 7 presents a gradient decline distribution of temperature. During this period, MCO-PINN can accurately predict the temperature of the workpiece, although both the PINN and FEM methods exhibit larger prediction errors—particularly for the FEM method, which struggles to provide effective predictions during non-steady-state thermal processes, resulting in substantial deviations from actual measured temperatures. However, as the temperature rises and furnace pressure increases, heated air gradually overflows from the bottom furnace door and the top crane suspension hole, slowing down the temperature rise. As the furnace approaches a near-vacuum state, the process transitions from rapid heating to the temperature holding period. The temperature profiles for each zone during the temperature transition period are also depicted in Figure 7a,b, where the maximum temperature increase within 100 s is less than 5 °C. Although the prediction errors for PINN and FEM slightly improve with reduced furnace temperature change rates, the MCO-PINN method still demonstrates the highest prediction accuracy for temperature. The experimental results demonstrate that the proposed MCO-PINN method maintains a high level of temperature prediction accuracy under complex heat exchange conditions, exhibiting robustness in its performance.

**Figure 8:**
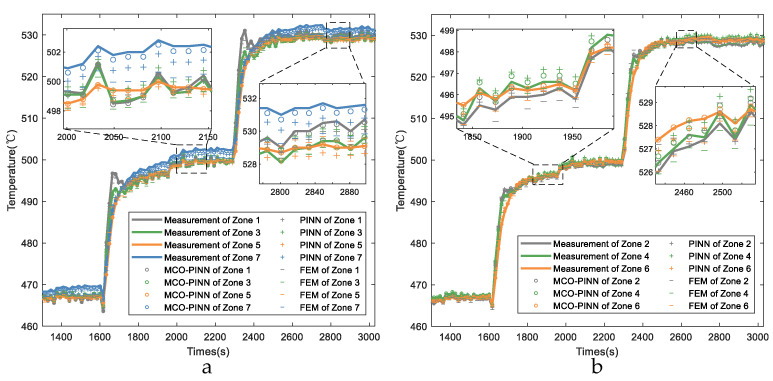
Comparisons of the workpiece temperature with different methods in the temperature holding periods (**a**) Zones 1, 3, 5, and 7. (**b**) Zones 2, 4, and 6.

Figure 8 presents the temperature profiles of the aluminum alloy workpiece calculated by different models during the gradient temperature holding period. According to the quenching process, to fully integrate the alloy components into the aluminum matrix during the temperature holding period, gradient temperature setpoints are set as 465 °C, 500 °C, and 530 °C for this specific type of workpiece. As shown in Figure 8, it can be found that during this temperature holding period, the furnace is primarily characterized by radiant heat exchange, with a relatively uniform heat exchange mode and stable furnace conditions. The maximum temperature difference across various zones within the furnace is maintained within ±3 °C. The MCO-PINN method continues to accurately track the measured workpiece temperatures. Although the prediction accuracy of the FEM method is better than that of non-steady heat transfer, the prediction accuracy of the FEM method is still inferior to that of the PINN method, and the MCO-PINN method is still the highest in precision.

**Figure 9:**
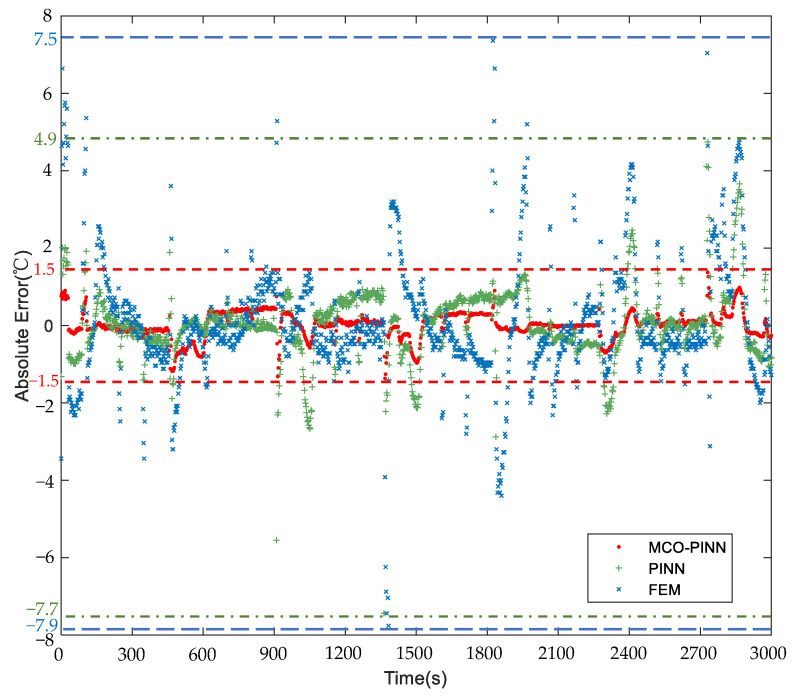
Comparisons of the absolute error of MCO-PINN, PINN, and FEM.

To clearly illustrate the prediction accuracy of different solution methods, Figure 9 displays the absolute error for each method. It is observed from Figure 9 that the prediction accuracy of MCO-PINN can be maintained within ±1.5 °C, satisfying the quenching process requirement of a ±3 °C uniform temperature distribution inside the workpiece. The prediction error range of FEM ranges from 7.5 °C to −7.9 °C, while the PINN method ranges from 4.9 °C to −7.7 °C, both failing to meet the ±3 °C uniform temperature distribution requirement. Moreover, large error fluctuations are observed during the rapid heating at 100 s and during the gradient heating at 1700 s and 2400 s within the temperature holding period. This result indicates that the FEM method is highly susceptible to changes in heat exchange conditions, resulting in unstable prediction accuracy. The PINN method also exhibits significant errors under unstable heat exchange situations. However, the MCO-PINN method demonstrates smaller fluctuations under these conditions. Therefore, the accuracy and stability of the MCO-PINN method are notably superior to those of the PINN and FEM methods.

**Figure 10:**
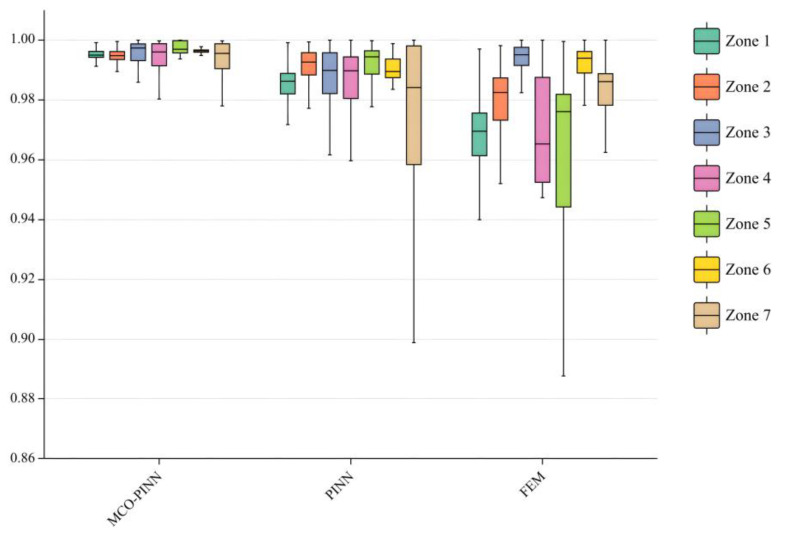
Box plot of prediction rates of MCO-PINN, PINN, and FEM.

Figure 10 presents boxplots illustrating the temperature prediction accuracies of MCO-PINN, PINN, and FEM methods. The temperature accuracy is evaluated by the R^2^ index. R^2^ (R-squared) is a measure to describe the degree of interpretation of independent variables to dependent variables in statistical models. The line within the box indicates the median of the data, while the box itself encloses 50% of the dataset, reflecting the fluctuation in prediction accuracy for each method. The upper and lower edges of the box represent the maximum and minimum values of the dataset, respectively. As depicted in Figure 10, the temperature prediction accuracy of MCO-PINN fluctuates between 0.98 and 1 across the seven zones, substantially outperforming the other methods. The height of the MCO-PINN box is relatively low, centered around 0.9, with the smallest difference between the upper and lower edges, indicating that its overall prediction accuracy is relatively stable. The FEM method exhibits the largest difference between its box edges, suggesting greater fluctuations in temperature prediction accuracy. Therefore, compared to PINN and FEM methods, the MCO-PINN method demonstrates superior recognition and stability. It maintains high computational accuracy throughout the entire thermal treatment process, even accurately predicting complex states with dramatic heat exchanges. This efficiency effectively provides rapid and reliable feedback information for controlling uniform temperature distribution in the quenching furnace, contributing to the improvement of aluminum alloy workpiece quality.

**Figure 11:**
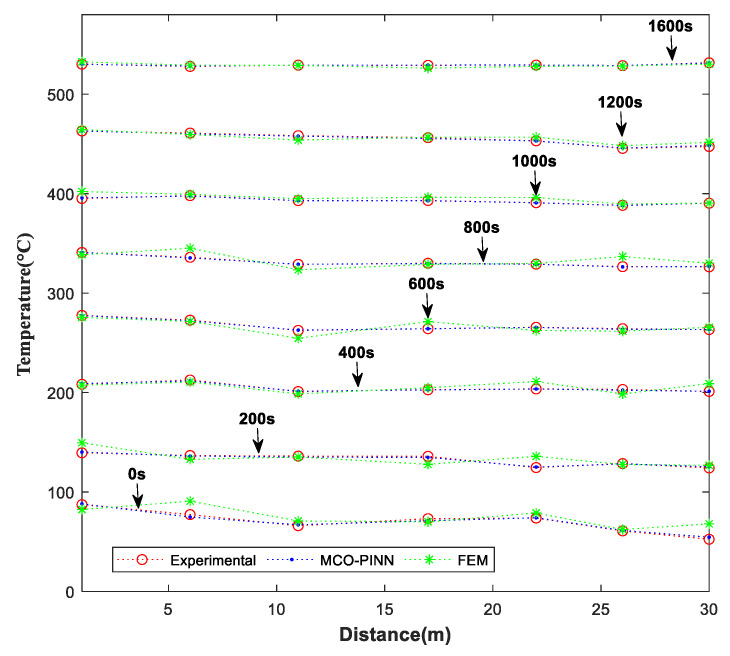
Comparisons of MCO-PINN and FEM at different times.

To further validate the performance of MCO-PINN in solving transient 3D temperature field models in the time dimension, Figure 11 displays the temperature curves of seven thermocouple measurement points on the workpiece over time, calculated using various methods. The workpiece extends 30 m in length, with the axis denoting position; 0 represents the installation position of Zone 1 thermocouple at the furnace top, and 30 corresponds to the installation position of Zone 7 thermocouple at the furnace bottom. The temperature distributions at different time steps for each thermocouple, calculated using MCO-PINN, PINN, and FEM, are shown in Figure 11. As illustrated in Figure 11, the MCO-PINN method exhibits high temperature prediction accuracy throughout the entire thermal treatment process, displaying the best alignment with the temperature values at the seven measurement points. The PINN and FEM methods show noticeable fluctuations in prediction accuracy during the temperature rising period, such as 200 s, 400 s, and 600 s, with the most significant temperature variations observed in Zones 1 and 7. Moreover, during high-temperature stages when the temperature exceeds 450 °C, both MCO-PINN and FEM methods achieve high-precision workpiece temperature predictions due to the stable heat exchange, with improved prediction accuracy compared to that of low-temperature stages. However, when temperatures fall below 450 °C, significant deviations between FEM predictions and actual temperature measurements are observed at the upper parts of the workpiece, such as positions 0 m and 6 m, as well as the lower sections at 21 m and 30 m. These locations, which are close to the top and bottom of the furnace, are more susceptible to external environmental influences. Nevertheless, MCO-PINN maintains high-precision temperature predictions for the workpiece, remaining virtually unaffected by the complexity of heat exchanges within the furnace and external environmental factors.

To comprehensively compare the computational performance of different solution methods, Table 1 provides statistical indicators of the performance obtained using FEM, PINN, and MCO-PINN. MRE and RRMSE denote the mean relative error and the relative root-mean-squared error, respectively. The 1.5% hit rate and 0.2% hit rate indicate that the relative error of temperature prediction during the temperature rising and holding periods is below 1.5% and 0.2%, respectively. The statistics reveal that the MRE range for FEM is 0.277–0.655, 0.151–0.332 for PINN, and 0.028–0.141 for MCO-PINN. Regarding RRMRE, the minimum value is 0.005 for FEM and 0.002 for PINN, but MCO-PINN has the lowest minimum value of 0.001, and its maximum is only 0.003. The 1.5% hit rate and 0.2% hit rate for MCO-PINN can reach 0.999 and 0.998, respectively, surpassing those of PINNs and the FEM. The number of iterations also indicates that the MCO-PINN iteration speed is considerably faster than PINNs and FEM. These data suggest that MCO-PINN not only has the highest computational accuracy but also boasts the fastest computation speed. Therefore, MCO-PINN can facilitate real-time, high-precision predictions of large-scale 3D temperature fields.

## 4. Discussion

In this study, we introduced the MCO-PINN for soft sensing of the 3D temperature field model of large aluminum alloy workpieces during quenching thermal treatment. A comparative analysis of MCO-PINN, traditional PINN, and FEM demonstrate the superior performance of the proposed technique in terms of accuracy and computational efficiency. Our results highlighted that MCO-PINN consistently outperformed the other methods in predicting the temperature distribution of the workpiece. Despite the complex heat exchange dynamics within the furnace and potential external factors, MCO-PINN maintained exceptional accuracy and stability. Furthermore, MCO-PINN demonstrated a notably faster iteration speed compared to both PINN and FEM, enabling real-time, high-precision predictions of large-scale 3D temperature fields. The effectiveness of the MCO-PINN method can be attributed to several factors. Firstly, the integration of a Gaussian probability distribution model into the loss functions of MCO-PINN accounts for the residuals of the PDE, initial and boundary conditions, and observed data, resulting in a substantially improved accuracy through the optimization of the weight consistency of each loss function. Secondly, the AIV-ECC algorithm facilitates the rapid determination of the Gaussian activation function parameters, minimizing the dependence on initial values and enhancing the generalization performance of the network. Despite the notable advantages of MCO-PINN, there are certain limitations of MCO-PINN. The study primarily focused on a specific shape of the workpiece during the quenching thermal treatment process, which might constrain its generalizability to other materials and thermal treatment conditions. Moreover, incorporating more experimental data could help validate the robustness of MCO-PINN in various practical applications. Future research directions could address these limitations by testing the performance of MCO-PINN with diverse materials and different shapes, particularly in industries where precise temperature control is critical. Moreover, using high-quality, diverse training data and labeled data can improve the model generalization ability. MCO-PINN has shown great promise in predicting the 3D transient temperature field of large aluminum alloy workpieces during the quenching thermal treatment process with impressive accuracy and computational efficiency.

## 5. Conclusions

This study presents an innovative soft-sensing method for high-dimensional, transient, and large-scale temperature field prediction of aluminum alloy workpieces based on the MCO-PINN model. A Gaussian radial-basis-function-based MLP network structure has been employed, integrating the constraints of PDE physics and observed data information into network loss functions. Through a multi-loss consistency optimization method, the effect of a weighted combination of competitive multiple-loss functions on network accuracy is reduced, resulting in improved precision of the network. Furthermore, the AIV-ECC algorithm has been introduced, accelerating network training speed and minimizing reliance on initial values. Compared to FEM and PINN methods, the proposed MCO-PINN approach demonstrates superior accuracy and time efficiency in predicting large-scale temperature fields, both in steady-state and complex scenarios with more intense heat exchange. The MCO-PINN method eliminates the complexity of numerical solutions for physical models and the dependence on extensive labeled data, enabling precise and rapid predictions based on sparse temperature measurements at designated locations. This innovative approach holds significant potential for accurately and efficiently predicting high-dimensional complex thermal treatment processes and large-scale scenarios in diverse fields, such as thermal treatment processes for slabs, glass, ceramics, semiconductor manufacturing, and large-scale chemical reactors. In these applications, obtaining accurate temperature field distributions and maintaining uniformity are crucial for enhancing material properties and optimizing product quality. Future research will focus on utilizing higher quality, diverse training datasets to further improve the generalization and predictive capabilities of the MCO-PINN method. Additionally, investigating transfer learning techniques will be explored to increase the model’s robustness across various materials and industrial processes, broadening the potential application of the MCO-PINN model.

## Figures and Tables

**Table 1:** Comparisons of statistical indexes of FEM, PINN, and MCO-PINN.

Indexes	Methods	Zone 1	Zone 2	Zone 3	Zone 4	Zone 5	Zone 6	Zone 7
MRE	FEM	0.477	0.358	0.378	0.423	0.527	0.277	0.655
	PINN	0.177	0.171	0.232	0.215	0.151	0.188	0.332
	MCO-PINN	0.061	0.141	0.061	0.099	0.028	0.066	0.108
RRMRE	FEM	0.010	0.014	0.015	0.010	0.012	0.005	0.023
	PINN	0.003	0.005	0.006	0.007	0.002	0.003	0.008
	MCO-PINN	0.001	0.003	0.002	0.002	0.001	0.001	0.003
1.5% hit rate	FEM	0.849	0.898	0.967	0.878	0.775	0.963	0.906
	PINN	0.968	0.974	0.963	0.961	0.987	0.957	0.928
	MCO-PINN	0.999	0.992	0.999	0.988	0.994	0.998	0.984
0.2% hit rate	FEM	0.875	0.850	0.920	0.872	0.813	0.866	0.802
	PINN	0.963	0.992	0.977	0.968	0.976	0.949	0.908
	MCO-PINN	0.998	0.994	0.987	0.987	0.994	0.968	0.969
Iterations	MCO-PINN	4943						
	FEM	18,891						
	PINN	9230						

## Data Availability

The data presented in this study are available on request from Z.C.
